# Effect of Extract-Added Water Derived from Deep-Sea Water with Different Hardness on Cognitive Function, Motor Ability and Serum Indexes of Obese Mice

**DOI:** 10.3390/nu14091794

**Published:** 2022-04-25

**Authors:** Koji Fukui, Yuki Suzuki, Yugo Kato, Nozomu Takeuchi, Hirotsugu Takenaka, Masahiro Kohno

**Affiliations:** 1 Molecular Cell Biology Laboratory, Department of Bioscience and Engineering, College of System Engineering and Science, Shibaura Institute of Technology, Fukasaku 307, Minuma-ku, Saitama 337-8570, Japan; bn16028@shibaura-it.ac.jp (Y.S.); nb19102@shibaura-it.ac.jp (Y.K.); kono.masahiro.d4@sic.shibaura-it.ac.jp (M.K.); 2 Dydo-Takenaka Beverage Co., Ltd., Haneyou Ko 1310-1, Muroto 781-6741, Japan; takeuchi@dt-beverage.com (N.T.); takenaka@dt-beverage.com (H.T.)

**Keywords:** deep-sea water, different hardness, obese mice, cognition, motor ability

## Abstract

Deep-sea water (DSW) contains multiple minerals and is widely used as drinking water, for cosmetic purposes, and as seasoning. In this study, several types of extract-added water with different levels of hardness (200, 300, 500) were prepared from DSW collected off the coast of Muroto City, Kochi Prefecture. We administrated it to obese mice for two months and tested it for several effects. Although there was no anti-obesity effect for any hardness level in obese mice, the cognitive functions of each DSW-extract-added water-treated group were significantly improved compared to control obese mice in the water maze test. Time-to-fall by the rota-rod test was also dramatically improved in the DSW-extract-added water-treated groups. The levels of triglycerides and blood urea nitrogen were significantly decreased in DSW-extract-added water-treated obese mice. However, these results did not depend on the hardness. Hardness levels of 200 or 300 of DSW-extract-added water had greater effects on cognitive function and serum scores compared to a level of 500. We analyzed DSW using inductively coupled plasma atomic emission spectroscopy and inductively coupled plasma mass spectrometry. High concentrations of magnesium and potassium were detected, but sodium was not detected at very high concentrations. Although the detailed mechanisms of its effects are not yet understood, chronic intake of DSW-extract-added water may have a beneficial effect on health.

## 1. Introduction

Over 95% of our body comprises carbon, hydrogen, oxygen, and nitrogen, and the remaining 4% is minerals [1,2]. Minerals are defined as essential elements not including carbon, hydrogen, oxygen, or nitrogen. Nutrients like protein, fats, carbohydrates, vitamins, and water are essential for our body to survive [3]. Each nutrient plays an important role in the maintaining vital activities, and minerals fall into two groups, major and trace elements. Major minerals include sodium, potassium, calcium, magnesium, phosphorus, sulfur, and chlorine, while trace minerals include iron, zinc, copper, manganese, selenium, chromium, molybdenum, cobalt, and iodine [4,5,6]. In Japan, the Ministry of Health, Labor, and Welfare reports dietary intake standards every year, which sets target daily intakes of various minerals by age [7]. For example, 3000 mg potassium, 800 mg calcium, and 340 mg magnesium are required per day in adult males in their twenties. Due to changes in social structure, lifestyle, and diet, it is doubtful that everyone takes in enough minerals. Chronic mineral deficiency has been reported to increase the risk of developing frailty syndrome in the elderly [8]. Therefore, many supplements are sold to the public, but there are many other ways to make up for the mineral deficiency. One solution is to drink natural mineral water, which is usually high in minerals such as calcium, potassium, and magnesium, so it supplements mineral intake. The size of the Japanese natural mineral water market size exceeds 300 billion yen [9], and consumption is increasing [10]. The consumption of bottled mineral water in 2020 was about 33.3 L/person/year [11]. This trend is expected to increase further due to growing health consciousness.

One beverage sold as a mineral supplement is deep-sea water (DSW). To be exact, it is sold as water to which a mineral extract obtained from DSW is added. DSW is characterized by a high mineral content of potassium, calcium, and magnesium, and high purity and, low temperature [12]. DSW, which is defined as seawater from a depth of 200 m or more, is used in various fields [13]. Recently, evidence has indicated that DSW has beneficial effects such as reducing obesity [14], cardiovascular disease risk [15], and blood pressure [16]. However, the detailed mechanisms underlying these functions are unknown. The problem with natural mineral waters including DSW-extract-added water is that they have different hardness and compositions. Therefore, it is difficult to compare the effects of each water. Water with too high of hardness is not suitable for daily intake and may adversely affect the body. In this study, to determine the most beneficial hardness, we fed a high-fat diet to C57BL/6 young mice and administrated DSW-extract water with different hardness values and evaluated effects on cognitive and coordinative functions and other biochemical and blood parameters. Based on the results of this experiment, we attempted to determine the biological effects and most beneficial hardness of DSW-extract-added water.

## 2. Materials and Methods

### 2.1. Animals

Before starting this study, all design of experiments was submitted and approved by the Animal Protection and Ethics Committee of Shibaura Institute of Technology (Approval number #19005, approval day 24 June 2019). Four-week-old C57BL/6J male mice were purchased from Sankyo Labo Service Corp. Inc. (Tokyo, Japan). Mice were randomly assigned to one of five dietary groups: control diet (Ctrl), high fat diet (HFD), HFD + supplemented with hardness 200 mg/L water (Ctrl + 200), HFD + supplemented with hardness 300 mg/L water (Ctrl + 300), and HFD + supplemented with hardness 500 mg/L water (Ctrl + 500). The different hardness waters were provided by Dydo-Takenaka Beverage Co. Ltd. (Kochi, Japan). HFD model mice were generated by feeding a diet (#D12492, Research Diets Inc., New Brunswick, NJ, USA) containing 5.24 kcal/g, with 60% of the calories from fat, 20% from protein, and 20% from carbohydrates to normal four-week-old mice until 12 weeks of age [17]. A control diet (CD) (#D12450J, Research Diets Inc.) was used for the control group, and the composition was 3.85 kcal/g, with 10% of the calories from fat, 20% from protein, and 70% from carbohydrates. The number of experimental animals is described in each figure’s caption.

The feeding period of these drinking waters was the same as with the HFD, until 12 weeks old. Mice were bred under conditions of a 12-h light/dark cycle and a temperature of 24 ± 2 °C. During the study period, all animals had free access to food and water. Food and water intake volumes and body weights were measured weekly. After the feeding period, we performed each behavioral test. The mice were anesthetized by ether and sacrificed quickly by decapitation, and the cerebral cortex, hippocampus, and liver were removed, and serum samples were taken. All chemical reagents were obtained from either FUJIFILM Wako Pure Chemical Industries, Ltd. (Osaka, Japan) or Sigma-Aldrich Corp. (St. Louis, MO, USA).

### 2.2. Water Preparation

DSW-extract-added waters of different hardness were made and provided by Dydo-Takenaka Beverage Co. Ltd. DSW was collected from offshore of Cape Muroto in Kochi Prefecture [13]. The extracts were concentrated through a filter, and the same lot of extracts were used to make different hardness of DSW-extract-added water (hardness 200, 300, and 500). The DSW extract was diluted with filtered well water. However, filtered tap water was administrated to the control and the HFD-treated groups. Details of the dissolved elements in each water are described in Table 1.

### 2.3. Behavioral Assessment

#### 2.3.1. Morris Water Maze

Cognitive function, especially learning ability was measured using a Morris water maze apparatus (#MWM-04M, Muromachi Kikai Co., Ltd., Tokyo, Japan) [18,19]. The apparatus (120 cm in diameter and 30 cm in height) consists of a pool constructed of vinyl chloride. The bottom of the pool was divided into four compartments by waterproof insulation tape, and four different visible marks (circle, triangle, square, and cross) were placed around the wall of the pool. The clear acrylic platform was placed in the center of one quadrant. The water temperature in the pool was maintained at 22 ± 2 °C. As an acclimation period prior to the cognitive test, the mice swam freely in the pool for 60 s without a platform for 3 days. The trials were performed four times per day for five consecutive days. All trials were performed at the same time of day and were carried out every 3 h (starting at 9:00, 12:00, 15:00, and 18:00). The position of the platform did not change was maintained in the same location of the pool for all trials (quadrant of “circle”). The time to reach the platform, swimming distance and speed, and the ratio of platform quadrant swimming time were measured using a behavior analysis software (ANY-maze, ver. 4.98; Stoelting Co., Wood Dale, IL, USA).

#### 2.3.2. Rota-Rod and Y-Maze Test

Motor coordination ability was determined by a rota-rod (#MK-670, Muromachi Kikai Co., Ltd.) test, as described previously, with some modifications [20]. The rod was accelerated from 5 to 50 rpm over a duration of 120 s. The time and rotation speed at the time of falling was recorded.

To the judgment of short-term memory, a Y-maze apparatus (Muromachi Kikai Co., Ltd.) was used [21]. The mouse was free to move in the maze for 10 min, and after shooting a video, the ANY-maze software was used to calculate the alternation behavior rate.

### 2.4. Western Blotting

Each brain region (cerebral cortex and hippocampus) was homogenized in RIPA buffer and used in western blotting as described previously [22], with some modifications. After concentration, protein volumes were measured using the Bradford assay (Bio-Rad protein assay, #500-0006JA, Bio-Rad Laboratories, Inc., Hercules, CA, USA) according to the manufacturer’s protocol. Twenty micrograms of protein extracts were run and separated on 12% SDS-polyacrylamide gels and transferred to cellulose nitrate membranes (ClearTrans, 0.2 μm). Two percent non-fat skim milk solution which is diluted by Tris-HCl-buffered saline (pH 7.6, containing 0.1% Tween 20 (TBS-T)) was used as a blocking solution, and the membranes were incubated for 1 h at room temperature. After washing the membranes with TBS-T, it was reacted with each primary antibody (anti-brain-derived neurotrophic factor (BDNF) (N-20) rabbit polyclonal antibody, 1:2500, #ab-108319 Abcam plc, Cambridge, UK; anti-nerve growth factor (NGF) (H-20) rabbit polyclonal antibody, 1:4000, #sc-548, SANTA CRUZ BIOTECHNOLOGY Inc., Dallas, TX, USA; anti-tropomyosin receptor kinase A (TrkA) (763), rabbit polyclonal antibody, #sc-118, SCBT Inc.; anti-TrkB (H-181) rabbit polyclonal antibody, #sc-8316, SCBT Inc.) overnight at 4 °C. Anti-rabbit IgG horseradish peroxidase-conjugated antibody (Promega Corp. Madison, WI) was used as secondary antibody at 1:4000 dilution for 1 h at room temperature. All signals were generated by incubation with the chemiluminescent reagents (Immobilon; Merck KGaA, Darmstadt, Germany). For normalization of the bands for each protein, the membranes were stained with Ponceau S solution (#24-3875-5, Merck KGaA). The relative intensities were determined using LAS-3000 (FUJIFILM Corp., Tokyo, Japan). Expression ratios were calculated by dividing each protein value by that of Ponceau S using ImageQuant TL software (ver.8.1, Global Life Sciences Technologies Japan, Tokyo, Japan).

### 2.5. Quantitative Analysis of DSW-Extract Added Water

The quantitative analysis of the metals contained in various waters was performed by inductively coupled plasma atomic emission spectroscopy (ICP-AES) (ICPS-8100, Shimadzu Corp., Kyoto, Japan) and inductively coupled plasma mass spectrometry (ICP-MS) (Agilent 7700, Agilent Technologies Japan, Ltd., Tokyo, Japan) by an external inspection agency (Shimadzu Techno-Research Inc., Kyoto, Japan).

### 2.6. Statistical Analysis

Data are expressed as means ± standard error (SE) and were analyzed using GraphPad Prism software (ver.9.0.0, GraphPad Software LLC., San Diego, CA, USA). *p*-values of less than 0.05 were considered statistically significant. The detailed statistical methods are described in the individual figure captions.

## 3. Results

### 3.1. DSW-Extract-Added Water Did Not Change Body Weights in HFD-Treated Mice

Mice were fed three different kinds of hard water and high-fat diets from 4 to 12 weeks of age. The body weights of all mice gradually increased in a time-dependent manner (Table 1A). The bodyweight of the HFD-treated group increased significantly compared to that of the age-matched control group from 5 to 8 weeks. Treatment with different hardness in the DSW-extract-added-water-treated group significantly increased body weight compared to those of the age-matched control group from 4 to 8 weeks. However, there were no significant differences in body weights due to the different hardness. There were no significant differences in food and water intake volumes among any groups (Table 1B,C).

### 3.2. DSW-Extract-Added Water Significantly Improved HFD-Induced Cognitive Dysfunction

**Figure 1:**
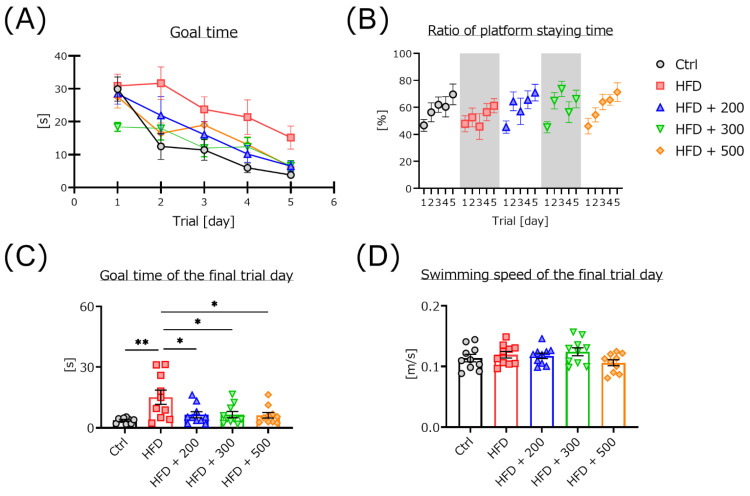
Differences in cognitive function between control, HFD-treated, and HFD plus DSW-extract-added water-treated mice. The time to goal (escape latency) in the Morris water maze test is shown in panel (**A**). The ratio of staying time in the platform quadrant is shown in panel (**B**). The average goal times of the final trial day are shown in panel (**C**). The average swimming speed on the final trial day is shown in panel (**D**). Control (Ctrl, *n* = 10), high-fat diet (HFD, *n* = 10), HFD + DSW-extract-added water hardness 200 (HFD + 200, *n* = 10), HFD + DSW-extract-added water hardness 300 (HFD + 300, *n* = 10), HFD + DSW-extract-added water hardness 500 (HFD + 500, *n* = 10). * *p* < 0.05, ** *p* < 0.01. The data are shown as means ± SE. Statistical analyses of the goal time were performed using two-way analysis of variance. Statistical analyses of the goal time for each day, swimming speed, and the ratio of staying time in the platform quadrant were performed using the Tukey-Kramer method.

To clarify the biological benefit of DSW-extract-added water, we measured cognitive function using the Morris water maze task. The daily average goal time gradually decreased in all mouse groups and confirmed that all mice learned the place of the escape platform (Figure 1A). The ratios of platform quadrant staying time were gradually increase of all mice (Figure 1B). However, the learning ability of the HFD-treated group was significantly lower compared to all other groups. The goal time of the HFD-treated mice showed significant delay compared to the controls. Treatment with different hardness of DSW-extract-added water significantly improved learning ability compared to the HFD-treated mice (Figure 1C). However, the average swimming speed did not change in any mouse groups (Figure 1D).

### 3.3. DSW-Extract-Added Water Significantly Improved HFD-Induced Coordination Disability

**Figure 2:**
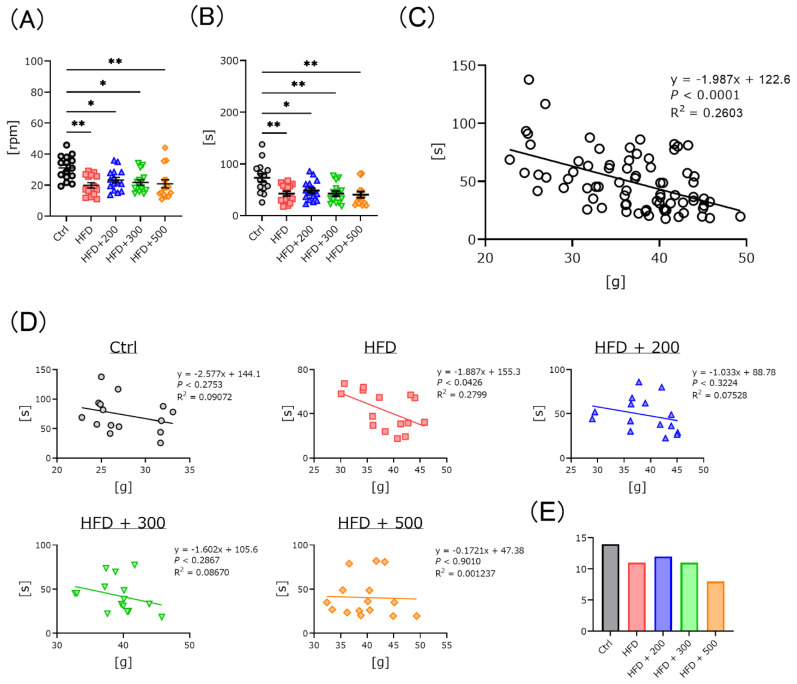
Time to fall in the rota-rod test. The fall speed and fall time are shown in panels (**A**) and (**B**). The relationship between weight and time to fall from the rod for all mice is shown in panel (**C**). The relationship between weight and time to fall from the rod for each mouse group is shown in panel (**D**). Panel (**E**) shows the number of mice that have remained on the rod for more than 30 s. Control (Ctrl, *n* = 15), high fat diet (HFD, *n* = 15), HFD + DSW-extract-added water hardness 200 (HFD + 200, *n* = 15), HFD + DSW-extract-added water hardness 300 (HFD + 300, *n* = 15), HFD + DSW-extract-added water hardness 500 (HFD + 500, *n* = 15). * *p* < 0.05, ** *p* < 0.01. The data are shown as means ± SE. Statistical analysis of data in panels A and B was performed using the Tukey-Kramer method. Those for panels (**C**) and (**D**) were performed using simple regression analysis.

DSW-extract contains many minerals. To further clarify DSW-extract-added water benefits, we measured mouse coordination ability using a rota-rod apparatus. Time-to-fall from the rod in HFD-treated mice was significantly faster than for the controls in the presence or absence of DSW-extract-added water (Figure 2A,B). Generally, time-to-fall from the rod depends on the body weight. We plotted body weights and time-to-fall from the rod of all mice, which showed a negative correlation (Figure 2C). Next, we plotted the relationship between body weight and time-to-fall from the rod for each group. The HFD-treated group showed a negative correlation, while the DSW-extract-added water-treated obese mice did not show a significant correlation for any of the different hardness groups (Figure 2D). We counted the number of animals that remained for more than 30 s walking on the rolling rod. The number of mice gradually decreased in a hardness-dependent manner (Figure 2E).

### 3.4. DSW-Extract-Added Water Did Not Change Exploratory Behavior in HFD-Treated Mice

**Figure 3:**
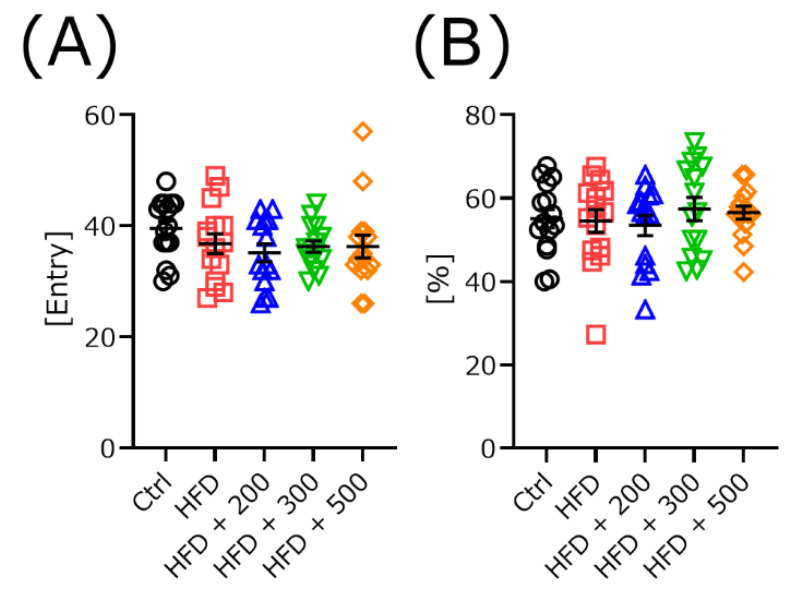
Total arm entries (**A**) and alternation scores (**B**) were measured using the Y-maze test. Control (Ctrl, *n* = 15), high-fat diet (HFD, *n* = 15), HFD + DSW-extract-added water hardness 200 (HFD + 200, *n* = 15), HFD + DSW-extract-added water hardness 300 (HFD + 300, *n* = 15), HFD + DSW-extract-added water hardness 500 (HFD + 500, *n* = 15). The data are shown as means ± SE. Comparisons were performed using the Tukey-Kramer method.

We measured the exploratory behavior using the Y-maze task. There were no significant differences in the number of arm entries (Figure 3A) and alternation scores (Figure 3B).

### 3.5. DSW-Extract-Added Water Did Not Change Neurotrophic Factor Secretion in HFD-Treated Mouse Brains

**Figure 4:**
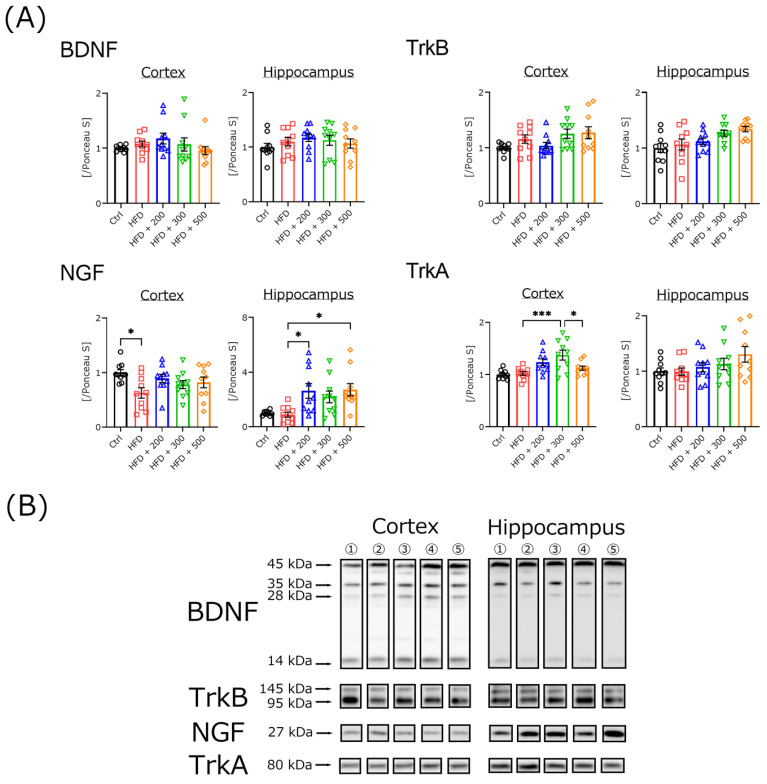
Western blotting analysis of the levels of neurotrophic factor-related proteins in the brains of control, HFD-treated, and HFD plus DSW-extract-added water-treated mice. All experiments were performed using cortex and hippocampal regions. The ratio of each protein band intensity to Ponceau S intensity is shown, with ratios of control samples set to 1 (**A**). Control (Ctrl, *n* = 10), high-fat diet (HFD, *n* = 10), HFD + DSW-extract-added water hardness 200 (HFD + 200, *n* = 10), HFD + DSW-extract-added water hardness-300 (HFD + 300, *n* = 10), HFD + DSW-extract-added water hardness 500 (HFD + 500, *n* = 10). * *p* < 0.05, *** *p* < 0.001. The data are shown as means ± SE. Comparisons were performed using the Tukey-Kramer method. Representative western blotting images (**B**).

Treatment with DSW-extract-added water reduced cognitive dysfunction in HFD-treated mice. To clarify the mechanism, after several maze tasks, we measured the expressions of neurotrophic factors such as NGF and BDNF and their receptors (Figure 4). NGF expression in the cerebral cortex of HFD-treated mice was significantly decreased compared to the controls. However, co-treatment with different water hardness did not decrease NGF expression in the cerebral cortex. Treatment with 200 or 500 hard water significantly increased hippocampal NGF expression in HFD-treated mice. TrkA, which is a receptor of NGF, was significantly increased in 300 hard-water-treated mice. However, the effect did not increase in a concentration-dependent manner.

### 3.6. Changes in Serum Parameters of DSW-Extract-Added Water-Treated Obese Mice

**Figure 5:**
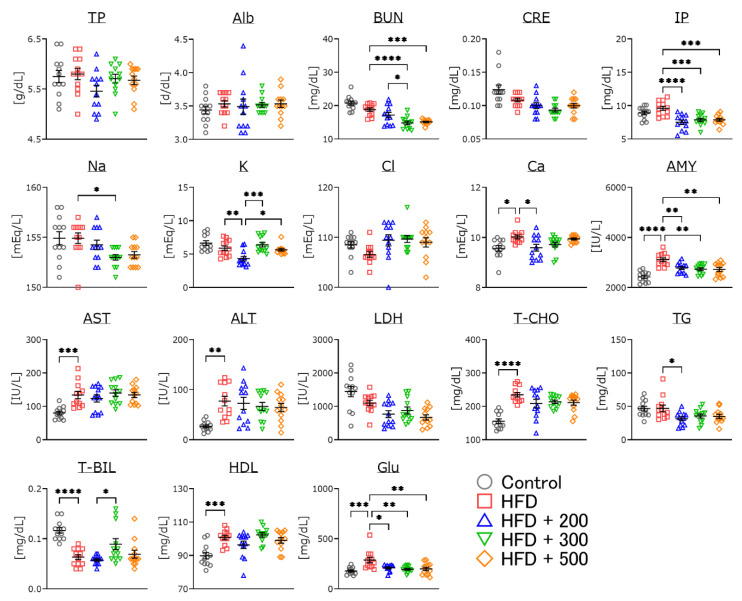
Changes in serum parameters for control, HFD-treated and HFD-plus DSW-extract-added water-treated mice. Control (Ctrl, *n* = 12), high fat diet (HFD, *n* = 12), HFD + DSW-extract-added water hardness 200 (HFD + 200, *n* = 12), HFD + DSW-extract-added water hardness 300 (HFD + 300, *n* = 12), HFD + DSW-extract-added water hardness 500 (HFD + 500, *n* = 12). The data are shown as means ± SE. * *p* < 0.05, ** *p* < 0.01, *** *p* < 0.001, **** *p* < 0.0001. Comparisons were performed using the Tukey-Kramer method. TP, total protein; Alb, albumin; BUN, blood urea nitrogen; CRE, creatinine; IP, inorganic phosphorus; Na, sodium; K, potassium; Cl, chlorine; Ca, calcium; AMY, amylase; AST, aspartate aminotransferase; ALT, alanine aminotransferase; LDH, lactic acid dehydrogenase; T-CHO, total cholesterol; TG, triglyceride; T-BIL, total bile acid; HDL, high density lipoprotein; Glu, glucose.

After several maze tasks, we measured serum parameters (Figure 5). Blood urea nitrogen, inorganic phosphorus, amylase, and glucose were significantly decreased in all DSW-extract-added water-treated groups compared to the HFD-treated mice. Calcium, amylase, aspartate aminotransferase, alanine aminotransferase, total cholesterol, total bile acid, high-density lipoprotein, and glucose were significantly increased in the HFD-treated group compared to the controls. Potassium, calcium, triglyceride, and total bile acid were significantly lower in hardness 200-treated water compared to the HFD-treated or other hard-water-treated groups.

### 3.7. DSW-Extract-Added Water Improved Fatty Liver but Not Liver Weight in HFD-Treated Mice

**Figure 6:**
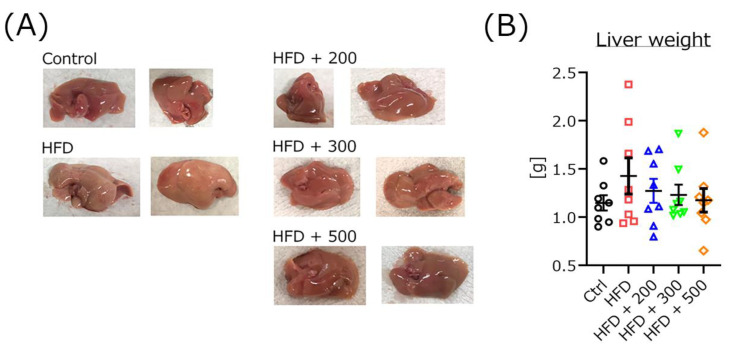
Livers removed after treatment. Liver images (**A**). Control (Ctrl), high-fat diet (HFD), HFD + DSW-extract-added water hardness 200 (HFD + 200), HFD + DSW-extract-added water hardness 300 (HFD + 300), HFD + DSW-extract-added water hardness 500 (HFD + 500). The liver weight of each mouse group (**B**). (*n* = 8, respectively) There were no significant differences among all groups. The data are shown as means ± SE. Comparisons were performed using the Tukey-Kramer method.

After the maze task, the livers were removed, and imaged (Figure 6A). Treatment with HFD resulted in mouse livers becoming increasingly white and congested. However, co-treatment with each DSW-extract added water remarkably improved the liver color similar to the original. There were no significant differences in the liver weight among all groups (Figure 6B) (Ctrl, 1.15 ± 0.08 g; HFD, 1.43 ± 0.19 g; HFD + 200, 1.27 ± 0.12 g; HFD + 300, 1.19 ± 0.10 g; HFD + 500, 1.18 ± 0.11 g).

### 3.8. Quantitative Analysis of the DSW-Extract-Added Water

Quantitative analysis of the metals contained in various waters was performed by ICP-AES and ICP-MS by an external inspection agency (Table 2). Since the lots of extracts derived from deep seawater with different hardness used in this study were all the same, only water with a hardness of 300 was analyzed. DSW-extract-added water (hardness 300) was diluted by filtered well water. The filtered well water did not contain any elements except a small volume of sodium. However, the DSW-extract-added water contained many elements such as calcium, magnesium, potassium, sodium, and other minor elements. Sodium levels only increased 2.5 times in DSW extract-added water (hardness 300) compared to the filtered tap water. In addition, the levels of potassium and magnesium in DSW-extracted added water (hardness 300) were more than five times greater than the filtered tap water. Elements not listed in the table were not detected in any of the samples.

## 4. Discussion

In this study, we evaluated the beneficial effects of DSW-extract-added water in obese mice. Some researchers have studied the biological functions of this water and have found that the main reason for its benefit is that it contains abundant minerals and is free of fats, proteins, and carbohydrates [12]. While similar studies have used only one water hardness, the novelty of this study was the use of three different water hardness (200, 300, and 500). In general, the hardness of tap water in Japan is from 50 to 80 [10]. Many mineral waters are sold on the private market, some with a hardness of over 500. Additionally, the hardness of tap water in some European countries is over 200 [10]. For these reasons, we believe that the hardness of our prepared DSW-extract-added water are not high.

In this study, we fed water of three different hardness to obese mice for two months. Water and food intake volumes were not significantly different among any mouse groups. These results indicate that there was no significant difference in the taste of water at different hardness and that it did not affect mice’s appetites. This result is supported by Hsu et al. [15], who administrated hardness 1500 DSW to HFD and high-cholesterol diet-treated hamsters for six weeks. They too found no significant differences in food and water intake volumes. Before starting this study, by administrating water with increasing hardness, we expected that obese mice would lose body weight over the two months. Contrary to expectations, there were no significant differences in body weights in obese mice with or without adding DSW-extract water (Table 1). We thought that certain minerals would help burn fat, but this study did not find such results. Some reports have found that treatment with DSW reduces body weight gain in HFD-treated C57BL/6 mice through the suppression of PPARγ expression [14,23]. However, in these reports, the hardness of the DSW used was 1000 or higher. There are also reports similar to ours showing that the body weight does not change [15,24,25]. Notably, the weights of the experimental animals in this study did not decrease due to diarrhea. The differences between studies may therefore depend on various factors such as the treatment period, hardness, month of age, and animal species. Furthermore, treatment with DSW is effective after hard training for fatigue recovery and attenuates oxidative stress [26,27,28]. Treatment with different hardness of DSW (300, 900, 1500) for six weeks significantly decreased lipid peroxidation products and increased glutathione levels in the livers of golden hamsters [25]. Considering that DSW is being taken continuously, there may be a synergistic effect when used in combination with other supplements, but this requires further study.

In the Morris water maze, the goal time gradually decreased in all mouse groups (Figure 1). The goal time for the HFD group was significantly lower than that for the controls. Co-treatment with DSW-extract significantly improved goal times for all three groups compared to the HFD group. However, the average swimming speeds on the final trial day were not significantly different in any of the mouse groups. Though we have found no articles directly studying cognitive function and DSW intake, there are many reports relating cognitive function and minerals such as zinc, magnesium, iron, and selenium [27,29]. Our results indicate that the reason for the low learning ability of HFD-treated mice is cognitive impairment and not motor dysfunction.

To clarify the coordination ability of the mice, the rota-rod test was used to measure the time to fall and the rod speed when the mice fell from the rod (Figure 2). Both scores were significantly lower for HFD-treated mice than those for the controls with or without DSW-extracted-added water. For a more detailed analysis of the data, the relationship between the time-to-fall scores and mouse body weights was plotted together for all groups. The time-to-fall scores significantly depended on body weight. Next, we examined the relationship between time-to-fall from the rod and body weight in each group. There were no significant differences in any groups except the HFD-treated group. These results indicate that treatment with DSW-extract water improved coordination in the HFD-treated mice compared to the untreated group. This may be related to the fatigue recovery effect [26]. Finally, we counted the number of animals that fell after more than 30 s. The hardness 200-treated group had the largest number among all groups. Although the body weights were large, there was a tendency for high scores in the 200 and 300 hardness administration groups. These results were not independent of hardness concentration. We also measured short-term memory and the alternation behavior rate using the Y-maze task (Figure 3). There were no significant differences for any groups. These results indicate that treatment with different water hardnesses did not affect these functions. The results were different for each behavioral task, so it is important to use several tasks to make a comprehensive judgment of the performance of each mouse groups.

To determine the mechanism of cognitive improvement in the Morris water maze and the rota-rod test, the expressions of neurotrophic factors (NGF and BDNF) and their receptors in the cerebral cortex and hippocampus were measured using western blotting (Figure 4). The expressions of BDNF and its receptor TrkB were not significantly different in any mouse groups. However, NGF expression in the cerebral cortex was significantly decreased in HFD-treated mice compared to the controls, and hippocampal NGF expressions in the groups with 200 and 500 water hardness were significantly increased compared to the HFD-treated group. TrkA, which is an NGF receptor, was significantly increased in the cerebral cortex of the 300 water hardness compared to the HFD-treated group. These results indicate that DSW-extract-added water induces neurotrophic factor expression in mouse brains.

There are several reports on the relationship between minerals and cognition, including for long-term, short-term, and working memory. Slutsky et al. reported that increasing the brain magnesium concentration enhances cognition via enhancement of synaptic plasticity in the hippocampal CA1 and DG region in Sprague Dawley rats [30]. The cellular zinc concentration is strongly related to the maintenance of axonal function [31], and potassium affects neuron-microglia interactions [32]. Although there are several lines of evidence indicating effects for each ion, the biological mechanisms of DSW-extracted-added water are not fully understood. They may be due to a mix of minerals or an unknown trace mineral, but further research is needed.

In the serum indexes, treatment with different hardness of DSW-extract-added water significantly decreased blood urea nitrogen and inorganic phosphorus (Figure 5). The creatinine score was nominally decreased (not significantly) in every DSW-extract-added water-treated group compared to the untreated group. These results indicate that treatment with DSW-extract-added water may affect renal function. AST, ALT, total cholesterol, and triglyceride were significantly increased in HFD-treated mice compared to the controls. However, DSW-extract-added water-treated mice did not show significant increases compared to the controls. These results indicate that DSW-extract-added water may also affect liver and cholesterol metabolism. Several lines of evidence have demonstrated that treatment with DSW (hardness 1000 or 1500) attenuates HFD-induced AST and ALT levels in rodent models [25,33]. In fact, in this study, the livers of HFD-treated mice clearly appeared dull and whitened, which was revered by DSW treatment (Figure 6). Some reports have shown that treatment with DSW decreases the liver size in HFD-treated rodent models [15,25]. Total bilirubin and amylase also were significantly changed in HFD-treated mice, and treatment with DSW-extract-added water improved these scores. These results indicate that DSW-extract-added water may affect pancreatic function. Finally, the serum glucose level of each DSW-extract-added water-treated group was significantly decreased compared to that of the HFD-treated mice. There are some reports indicating that treatment with DSW provides cardiovascular and arteriosclerosis protection via the attenuation of inflammation and cholesterol levels [15,28]. In short, treatment with DSW-extract-added water may affect hepato-biliary-pancreatic functions, though some reports have pointed out that DSW upregulates hepatic LDL receptor and cholesterol-7-α-hydroxylase gene expressions [15,34]. However, the detailed mechanisms are not yet fully understood. Further investigation is needed to clarify the relationship between inorganic ions and changes in serum indexes.

To clarify the beneficial functions of DSW-extract-added water, an elemental quantitative analysis (hardness 300) was performed using ICP-MS and ICP-AES. Twenty-one elements were detected in hardness 300 water with DSW-extract. The magnesium and potassium levels of the DSW-extract-added water were 11 and 7 times greater than filtered tap water. Generally, tap water in European countries is harder and contains calcium and magnesium, but our DSW-extract-added water was not high in calcium. However, the sodium level for the DSW-extract-added water was only 2.5 times more than the controls. In general, it is important to keep the increase in sodium ion concentrations low when concentrating DSW. Serum sodium ion concentrations in each group of DSW-extract-added water-treated mice were not increased compared to the two untreated groups. Interestingly, treatment with hardness 300 water resulted in significantly decreased serum sodium levels compared to the HFD-treated group. It is well known that high sodium levels are a high-risk factor for diabetes [35], high blood pressure [36], and cardiovascular risk [37]. Sodium levels in each water supplemented with the DSW-extract used in this experimental model should not have a significant effect on mice.

## 5. Conclusions

In this study, we determined the biological effects of different levels of hardness of DSW-extract-added water on obese mice. Several effects were not concentration dependent. In our model, hardness 200 or 300 may be the best for chronic intake based on the experimental data. Recently, due to changes in lifestyle, the intake of minerals has become insufficient, and the mineral balance in the body has been disturbed. Continued intake of beverages containing moderate mineral levels may help maintain proper health.

## Figures and Tables

**Table 1:** The effects of different hardness of DSW-extract-added water in mice.

(A)	Body Weight										
		0	1	2	3	4	5	6	7	8	[Week]
	Ctrl	18.97 ± 0.90	21.64 ± 0.87	23.18 ± 0.87	24.81 ± 1.06	25.83 ± 1.08	26.86 ± 1.13	27.40 ± 1.14	27.42 ± 1.01	27.84 ± 0.90	
	HFD	18.73 ± 0.74	22.74 ± 0.68	24.79 ± 0.76	27.19 ± 1.01	29.74 ± 1.08	32.11 ± 1.26 *	34.45 ± 1.35 **	36.80 ± 1.34 ***	38.46 ± 1.39 ***	
	HFD + 200	18.80 ± 0.86	23.08 ± 0.64	25.37 ± 0.86	27.94 ± 1.04	30.69 ± 1.13 *	33.29 ± 1.33 **	36.02 ± 1.47 ***	37.92 ± 1.46 ***	39.69 ± 1.46 ***	
	HFD + 300	18.45 ± 0.73	22.65 ± 0.35	25.06 ± 0.52	27.61 ± 0.64	30.16 ± 0.66 *	32.56 ± 0.73 **	35.54 ± 0.77 ***	37.89 ± 0.93 ***	39.56 ± 0.96 ***	
	HFD + 500	19.05 ± 0.87	23.27 ± 0.75	25.86 ± 0.98	28.41 ± 1.13	30.55 ± 1.12 *	33.04 ± 1.34 *	35.33 ± 1.34 **	37.06 ± 1.36 ***	38.98 ± 1.46 ***	[g]
(B)	Food Intake Volume										
		0	1	2	3	4	5	6	7	8	[Week]
	Ctrl	3.06 ± 0.08	2.66 ± 0.14	2.94 ± 0.15	2.70 ± 0.13	2.71 ± 0.08	3.03 ± 0.09	2.89 ± 0.18	3.10 ± 0.16	2.98 ± 0.15	
	HFD	3.31 ± 0.16	2.49 ± 0.13	2.70 ± 0.15	2.74 ± 0.13	2.71 ± 0.11	2.74 ± 0.10	2.98 ± 0.12	2.89 ± 0.12	3.05 ± 0.09	
	HFD + 200	3.33 ± 0.20	2.59 ± 0.11	2.59 ± 0.09	2.69 ± 0.09	2.74 ± 0.14	2.92 ± 0.08	3.01 ± 0.10	3.05 ± 0.14	3.04 ± 0.09	
	HFD + 300	3.32 ± 0.13	2.49 ± 0.13	2.72 ± 0.10	2.69 ± 0.11	2.69 ± 0.12	2.94 ± 0.11	2.95 ± 0.08	2.84 ± 0.13	3.19 ± 0.07	
	HFD + 500	3.41 ± 0.20	2.60 ± 0.11	2.64 ± 0.09	2.66 ± 0.08	2.75 ± 0.08	2.94 ± 0.08	3.03 ± 0.11	2.91 ± 0.11	3.07 ± 0.05	[g]
(C)	Water Intake Volume										
		0	1	2	3	4	5	6	7	8	[Week]
	Ctrl	3.25 ± 0.29	3.18 ± 0.25	3.26 ± 0.36	2.79 ± 0.20	2.84 ± 0.26	2.84 ± 0.24	3.11 ± 0.32	3.03 ± 0.22	3.06 ± 0.16	
	HFD	3.14 ± 0.21	2.87 ± 0.15	3.04 ± 0.23	2.86 ± 0.15	3.39 ± 0.39	2.86 ± 0.19	3.07 ± 0.23	3.04 ± 0.27	2.83 ± 0.18	
	HFD + 200	2.72 ± 0.13	2.61 ± 0.17	2.51 ± 0.16	2.69 ± 0.19	2.74 ± 0.16	2.46 ± 0.11	2.59 ± 0.16	2.96 ± 0.34	2.65 ± 0.21	
	HFD + 300	2.76 ± 0.12	2.74 ± 0.17	2.64 ± 0.14	2.79 ± 0.17	2.79 ± 0.15	2.56 ± 0.14	2.79 ± 0.18	3.01 ± 0.28	2.76 ± 0.17	
	HFD + 500	2.93 ± 0.10	2.84 ± 0.15	2.69 ± 0.13	2.83 ± 0.21	3.10 ± 0.27	2.88 ± 0.17	3.14 ± 0.28	3.04 ± 0.29	2.91 ± 0.26	[mL]

Increase of average weight of each mouse group from 4 to 12 weeks of age (A). Food and water intake for each mouse group (B, C). Control (Ctrl, *n* = 14), high-fat diet (HFD, *n* = 14), HFD + DSW-extract-added water hardness 200 (HFD + 200, *n* = 14), HFD + DSW-extract-added water hardness 300 (HFD + 300, *n* = 14), HFD + DSW-extract-added water hardness 500 (HFD + 500, *n* = 14). * *p* < 0.05, ** *p* < 0.01, *** *p* < 0.001 vs. same week Ctrl. The data are shown as means ± SE. Data were analyzed using a two-way analysis of variance.

**Table 2:** Quantitative analysis of elements contained in each water.

Element		Estimated Concentration [ng/mL]			Lower Limited
		Filtered Tap Water from Saitama City	Filtered Well Water from Kochi	H300 DSW Extract-Added Water *	[ng/mL]
Li	Litium	N.D.	N.D.	10	5
B	Boron	80	N.D.	200	10
Na	Sodium	20,000	500	50,000	200
Mg	Manesium	6000	N.D.	70,000	10
Si	Silicon	8000	N.D.	70	50
S	Sulfer	10,000	N.D.	300	100
K	Potassium	3000	N.D.	20,000	2000
Ca	Calcium	30,000	N.D.	20,000	10
V	Vanadium	0.7	N.D.	N.D.	0.1
Cr	Chromium	0.1	N.D.	0.1	0.1
Mn	Manganese	N.D.	N.D.	0.1	0.1
Fe	Iron	0.3	0.5	0.1	0.1
Ni	Nickel	0.8	N.D.	0.6	0.1
Cu	Copper	0.6	N.D.	2	0.1
Zn	Zinc	4	N.D.	2	0.5
Ga	Gallium	2	N.D.	0.2	0.1
Ge	Germanium	N.D.	N.D.	0.6	0.2
As	Arsenic	0.4	N.D.	N.D.	0.2
Br	Bromine	30	N.D.	4000	5
Rb	Rubidium	3	N.D.	6	0.1
Sr	Strontium	100	N.D.	300	10
Mo	Molybdenum	0.9	N.D.	N.D.	0.1
Sb	Antimony	0.1	N.D.	N.D.	0.1
I	Iodine	9	N.D.	4	1
Cs	Cesium	0.1	N.D.	N.D.	0.1
Ba	Barium	10	N.D.	0.8	0.1

(*) Hardness 300 deep sea water (DWS) Extreact-added water. DSW extract is diluted by filterd well water from Kochi City.
